# Impaired RNA incorporation and dimerization in live attenuated leader-variants of SIV_mac239_

**DOI:** 10.1186/1742-4690-3-96

**Published:** 2006-12-21

**Authors:** James B Whitney, Mark A Wainberg

**Affiliations:** 1 McGill University AIDS Centre, Lady Davis Institute-Jewish General Hospital, Montreal, Quebec, H3T 1E2, Canada; 2 Department of Microbiology and Immunology, McGill University, Montreal, Quebec, H3A 2B4, Canada; 3 Division of Viral Pathogenesis, Beth Israel Deaconess Medical Center, Harvard Medical School, Boston, MA 022115, USA

## Abstract

**Background:**

The 5' untranslated region (UTR) or leader sequence of simian immunodeficiency virus (SIV_mac239_) is multifunctional and harbors the regulatory elements for viral replication, persistence, gene translation, expression, and the packaging and dimerization of viral genomic RNA (vRNA). We have constructed a series of deletions in the SIV_mac239 _leader sequence in order to determine the involvement of this region in both the packaging and dimerization of viral genomic RNA. We also assessed the impact of these deletions upon viral infectiousness, replication kinetics and gene expression in cell lines and monkey peripheral blood mononuclear cells (PBMC).

**Results:**

Regions on both sides of the major splice donor (SD) were found to be necessary for the efficiency and specificity of viral genome packaging. However, stem-loop1 is critical for both RNA encapsidation and dimerization. Downstream elements between the splice donor and the initiation site of SIV-Gag have additive effects on RNA packaging and contribute to a lesser degree to RNA dimerization. The targeted disruption of structures on both sides of the SD also severely impacts viral infectiousness, gene expression and replication in both CEMx174 cells and rhesus PBMC.

**Conclusion:**

In the leader region of SIV_mac239_, stem-loop1 functions as the primary determinant for both RNA encapsidation and dimerization. Downstream elements between the splice donor and the translational initiation site of SIV-Gag are classified as secondary determinants and play a role in dimerization. Collectively, these data signify a linkage between the primary encapsidation determinant of SIV_mac239 _and RNA dimerization.

## Background

The 5' untranslated region (UTR) or leader sequence of lentivirus possess multiple structural and functional domains that include the regulatory elements for the initiation of reverse transcription, integration of the proviral genome, the trans-activation of RNA transcription, and protein translation. This region is also critical for both the encapsidation and dimerization of viral genomic RNA (vRNA). These latter functions act through *cis*-acting signals that are found within conserved structural domains of the viral leader sequence [1-3].

In regard to vRNA packaging in human immunodeficiency virus type-1 (HIV-1), the primary encapsidation determinant or Psi core (Ψ) has been shown to be located downstream of the major splice donor (SD), involving those structures that form stem loop-3 (SL3) and SL4 [4,5]. Upstream regions also have been described for their role in RNA encapsidation, particularly SL1 and the R and U3 regions [6]. The efficient packaging of vRNA also requires multipartite RNA-protein interaction of the leader RNA with the viral nucleocapsid (NC) domains of the Gag precursor (Pr^55^Gag), [7-9]. Interestingly, while HIV-1 has been shown capable of packaging the genomic RNA of either human immunodeficiency virus type-2 (HIV-2) or simian immunodeficiency virus (SIV) [10,11], the converse is not true, highlighting the important differences in the mechanism of RNA selection [10,12].

Although broadly comparable RNA secondary structures have been predicted for the leader regions of HIV-1, HIV-2 and SIV, prominent variations in sequence and structure are evident. For example, the sequences upstream of stem-loop 1 (SL1) in HIV-1 are 59 nucleotides long, whereas the comparable region in SIV_mac239 _is 38 nucleotides longer [13]. Similarly, the region downstream of the major SD of SIV_mac _is longer than the equivalent region of HIV-1. Functionally, this region in SIV has been shown to have internal ribosome entry site (IRES) activity, whereas, in the case of HIV-1, this element includes a region encompassing both sides of the SD [14,15]. Interestingly, HIV-1 has been shown to also encode an IRES element within the 5'*gag*-coding region [16].

Contrary to that described for HIV-1, the primary packaging locus (or Ψ core) of HIV-2 is located upstream of the SD [17,18]. In SIV, work by our group and others have described the regions 5' of the SD as important in encapsidation [13,19,20]. However, Poeschla *et al*. showed in HIV-2 that a large deletion adjacent to the 3' SD can abrogate encapsidation [21]. Griffin *et al *demonstrated that HIV-2, employs a co-translational mechanism of RNA packaging whereby newly translated Gag polyprotein interacts with vRNA while in a ribosomal translation complex, thereby imparting packaging specificity [22].

Implicit in the encapsidation process of all retroviral lineages except the Spumoretroviruses, is the formation of a linked RNA duplex or dimer consisting of two full-length vRNA molecules. Early in viral assembly, two copies of the viral genome bind at their 5' ends through non-covalent interaction of complimentary sequences located at the terminal palindrome of stem-loop 1 (SL1) [23]. This sequence has been aptly termed the dimerization initiation site (DIS) or kissing-loop domain (KLD) in the case of HIV-1 [24,25]. The association of viral RNA with NC protein catalyses its transition from a "immature" dimer into an extended "mature" state, termed the dimer linkage structure (DLS), concurrent with the maturation of nascent virions [26].

The importance of RNA-RNA interactions in selective genome packaging is based on evidence from the leader region of HIV-1 suggesting that SL1 alone is sufficient to initiate formation of this duplex, and enhances the rate of dimerization *in vitro *[27]. Forced evolutionary studies have revealed interaction of the DIS/KLD with multiple protein domains of NC as well as other regions of the Gag-polyprotein [28]. Genome packaging is also dependent on leader-RNA tertiary conformation. For example, *in-vitro *studies with HIV-2 describe alternating RNA structures that regulate both RNA packaging and dimerization [29-33]. However, the relationship between RNA packaging and RNA dimerization is still questioned ; some groups have proposed that different RNA structures may lead to alternate forms of interaction [34,35]. Others have suggested that differences among various retroviral systems may exist, lending a uniqueness to individual viruses or groups of viruses [36].

Our group has previously described the upstream SIV_mac239 _leader and its impact upon viral replication and RNA encapsidation [13,19]. In the present study, we have extended our analysis of several attenuated leader variants of SIV_mac239 _to include the regions on either side of the major SD, as no studies to date have investigated both RNA encapsidation and RNA dimerization in SIV. We show that elements on both sides of the major SD impact both RNA packaging and dimerization, and provide evidence of secondary packaging elements. We have also related deficits in RNA packaging and dimerization to overall viral infectivity and replication capacity.

## Results

### RNA structural domains on both sides of the major splice donor regulate the efficiency and specificity of SIV_mac239 _RNA packaging and dimerization

**Figure 1:**
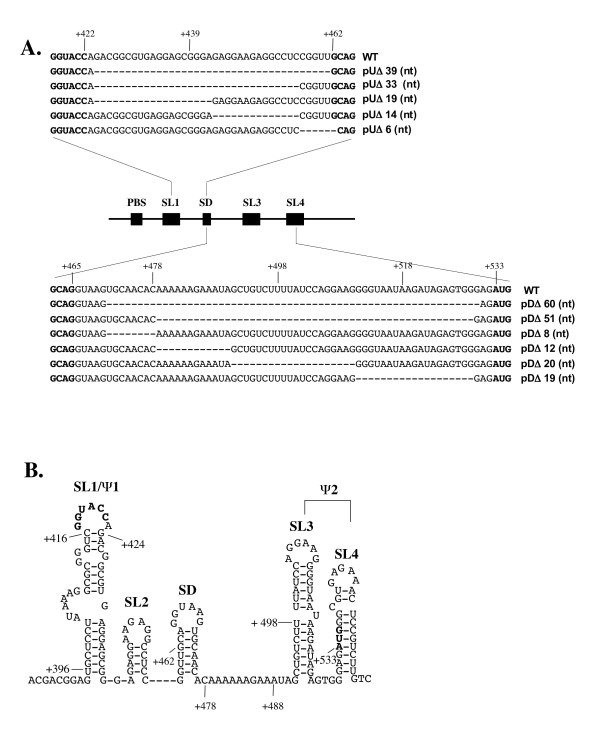
**Nucleotide position of deletion mutations within the leader sequence and the secondary structure of SIV_mac239 _leader RNA**. **A**. Denotes the size and nucleotide position of deletion mutations located upstream (**pU**) or downstream (**pD**) of the major SD. All nucleotide deletions are relative to the transcriptional initiation site (1+) based on the sequence of the wild type clone of SIV_mac239 _[50]. **B**. Secondary structure was adapted from published information [13, 51]. All hairpin motifs are labeled according to their putative function and/or after comparable elements encoded by HIV-1/HIV-2 leader sequences. The following motifs are shown in bold type: the putative DIS palindrome at position +417-422, the splice donor (SD) at position +462, and the Gag initiation codon at position +533.

**Figure 2:**
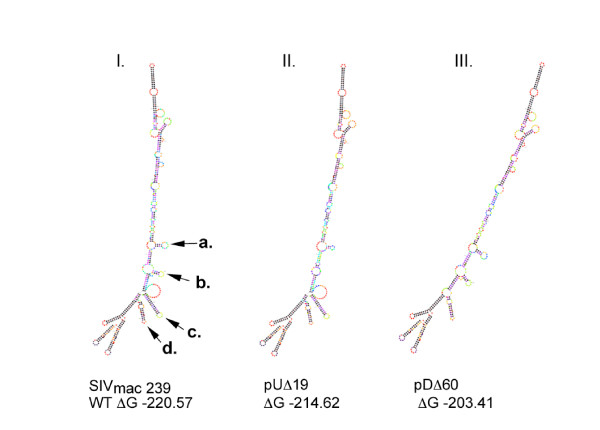
**Thermodynamic folding analysis of resolved RNA SIV_mac239 _leader structures**. Shown are predicted free-energy minimized structures from the SIV_mac239 _wild-type leader (I.) and attenuated mutants pUΔ19 (II.) and pDΔ60 (III.) [51]. Fig. I, arrows labeled **a**, **b**, **c**, and **d **denote SL1, SL2, SL3 and SL4, respectively, in the SIV_mac239 _leader structures or their absence in deletion variants.

We have constructed a series of proviral mutants within the leader of SIV_mac239 _(Fig. 1A and 1B) and have assessed vRNA packaging efficiency through both replicate slot blotting and multiplex RT-PCR experiments. We have also determined the impact of these mutations on vRNA dimerization by recovery of RNA from mature virions and analysis on native Northern gels. A diagrammatic depiction of the folding of several of these RNA structures is shown in Fig 2.

#### RNA packaging

**Figure 3:**
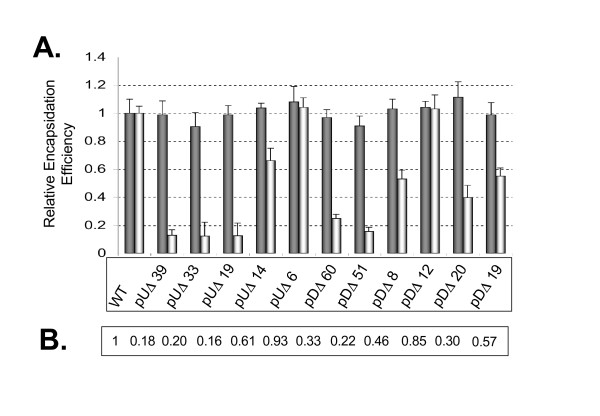
**Both genomic RNA packaging efficiency and specificity are compromised in SIV_mac239 _by mutations within the leader region**. **A**. Analysis of viral RNA packaging was conducted in triplicate in COS-7 cells. Shown are results from purified viral RNA preparations that were normalized on the basis of p27-CA ELISA. RNA packaging of various mutant constructs as a percent average of WT virus. The bars of the first lane for each sample represent total incorporated viral RNA. The second bar for each lane represents incorporated full-length viral RNA. **B**. Shown at the bottom is percent specific incorporation as a ratio of that obtained with the wild type full-length genome.

The results of Fig. 3 show the average of three slot-blotting experiments conducted with viral supernatants normalized on the basis of p-27CA content. Slot blots were probed with radioactively labeled probes to measure either full-length or total RNA content from lysed virions. Relative RNA encapsidation was determined and by amounts were quantified by molecular imaging. Multiplex RT-PCR as described previously [[13], see also Materials and Methods], was also used to corroborate slot-blotting data (not shown). The largest reduction in packaging efficiency was observed in the constructs that removed or disrupted the distal stem portion of SL1 (i.e. mutants pUΔ39, pUΔ33, and pUΔ19). This RNA-deficient phenotype was localized to the nucleotide region of SL1 removed in pUΔ19 (Fig 1. and 3A) as deletion of the regions directly adjacent (i.e. pUΔ14 and pUΔ6) resulted in modest or no significant decrease in packaging, respectively. Analysis of RNA secondary structures was conducted using M-Fold software [48] and indicated that the pUΔ39 and pUΔ33 deletions resulted in abolition of both SL1 and SL2. However, for mutant pUΔ19, a more conservative refolding resulted in the loss of the GGUACC DIS sequence from the apical loop of SL1 and loss of the SL2 structure (Fig. 2).

We also conducted deletion analysis of RNA structural domains downstream of the SD. The largest deletions (i.e. pDΔ60 and pDΔ51) resulted in severely diminished packaging. Thermodynamic analysis of these mutants was also conducted using M-Fold software and revealed a loss of the SL3 element and refolding of the SD loop, but conservation of SL4 and the structural elements upstream of the SD, even though the deletions were quite large (Fig. 2, panel III). Packaging deficits through smaller deletions mutants could then be localized to SL3 and SL4; the impact of these deletions on RNA encapsidation was additive (Fig. 3).

We also considered the impact of these deletions on the specificity of RNA encapsidation by quantifying vRNA versus total encapsidated RNA ratios from virions recovered from cell-free supernantants (Fig 3B). Relative packaging efficiencies shown in Fig. 3 indicate that the total amount of RNA incorporated into each mutant virus was generally conserved; presumably these mutants also incorporated significant amounts of spliced viral RNA.

To ensure that packaging deficits were not the result of diminished availability of cytoplasmic vRNA, we also considered cell-associated vRNA concentrations. Towards this end, total RNA was recovered from lysates of transfected COS-7 cells and normalized on the basis of p27-capsid Ag present in cellular lysates. All experiments used the same probe that measured full-length vRNA as in slot-blotting analysis (see Materials and Methods). The cytoplasmic viral RNA levels in each case did not significantly deviate from those observed with wild-type virus (not shown).

#### RNA dimerization

**Figure 4:**
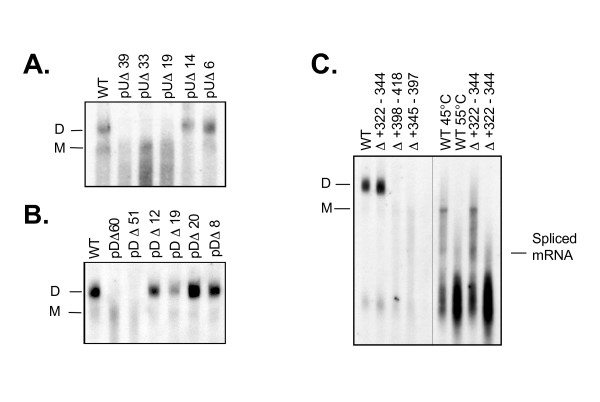
**Non-denaturing Northern analysis of SIV RNA**. Non-denaturing analysis of intra-virion RNA from transient transfections of mutant clones. Viral genomic RNA was isolated from virus particles after transfection of COS-7 cells with wild type or mutant plasmids. The relative mobility of dimers (D) and monomers (M) in 0.90% agarose are indicated. The plus (+) denotes the addition of RNase to preparations prior to electrophoresis. **A**. Shows non-denaturing RNA preparations from deletion mutants encompassing the region from nt +426 - +465. **B**. Shows non-denaturing RNA preparations from mutants encompassing the nt region +473 - +480. C. RNA preparations from mutants encompassing the region directly adjacent to the PBS, i.e. SD1, SD2, and SD3 deleted the of nucleotide regions +322-344, +398-418, and +345-397 respectively [13]. The adjacent panel shows thermal denaturing analysis of the SD1 mutant (Δ+322 - +344), comprising a 23-nucleotide deletion upstream of SL1 in comparison to dimer extracted from the wild-type virions.

As mentioned above, we also analyzed phenol-chloroform extracted vRNA by non-denaturing Northern analysis for each of the viral mutants from this study to determine their ability to incorporate viral RNA as a dimer. (Fig. 4A) shows the relative contribution of nucleotide sequences upstream of the major SD. For each of the pUΔ39, pUΔ33, and pUΔ19 mutants, no dimeric RNA was present, even after the overloading (4×) of viral RNA preparations onto non-denaturing gels. Of the smaller deletions, both pUΔ14 and pUΔ6 were capable of dimer formation.

The analysis of regions downstream of the major SD showed that only the largest deletions, i.e. pDΔ60 and pDΔ51, completely disrupted dimerization (Fig 4B). In contrast, viral RNA recovered from most constructs harboring smaller intermediate deletions within this region did not differ significantly from the wild-type dimer. One exception was mutant pDΔ19, which consistently showed a reduction in the amount of packaged dimeric RNA.

*In vitro *work from other groups has implicated regions upstream of SL1 as involved in the dimerization of HIV-2 RNA [31-33]. To examine this issue in SIV, we analyzed three mutants employed in previous studies (i.e. SD1, SD2, and SD3) deleted the of nucleotide regions +322-344, +398-418, and +345-397 respectively [13]. Analysis of purified RNA from the SD1 mutant (nt +322 to +344 directly adjacent to the PBS), showed no qualitative or quantitative differences in RNA dimer formation, mobility, or encapsidation compared to wild-type virus in either its native conformation or heat-denatured state (Fig. 4C).

### Deletion of regions implicated in viral RNA packaging and dimerization result in diminished infectivity and viral spread that correlates with aberrant viral core morphology

**Figure 5:**
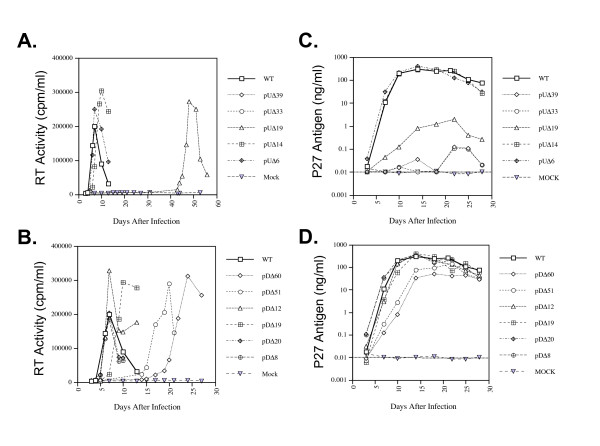
**Replication kinetics of various mutant constructs in CEMx174 cells and primary rhesus PBMC**. Cells were infected with 10 ng viral equivalents and viral replication was monitored by RT assay of culture supernatant at multiple time points. Mock denotes infection with heat-denatured wild-type virus. All replication experiments were conducted in triplicate. **A**. Representative growth curves of viruses deleted between the DIS and the SD (i.e. nt +424-462) in CEMx174 cells. **B**. Replication of viruses deleted between the SD and the Gag AUG (nt +471-530) in CEMx174 cells. Viral replication was assessed in activated rhesus PBMCs using viral inocula normalized on the basis of p27-CA Ag. Growth curves were determined by p27-CA Ag ELISA of culture supernatant taken at multiple time points. All results are the average of duplicates. **C**. Growth curves of variants deleted within the nt region +424-462. **D**. Growth curves of variants deleted within the region +471-530. Mock infection denotes exposure of cells to heat-inactivated wild-type virus as a negative control. Note that the scales of the ordinates are logarithmic. The dashed line representing 0.01 ng of p27/ml indicates the threshold of sensitivity of the assay.

The results of Fig. 5 show that all mutants displayed some replication impairment in the CEMx174 cell line as measured by RT assay. Specifically, mutants' pUΔ39, pUΔ33, and pUΔ19 showed severely impaired replication. Over 6 months of extended culture, both the pUΔ39 and pUΔ33 mutants consistently scored negative by RT assay, indicating that deletions within this region presented an insurmountable barrier to the recovery of productive viral replication. The pUΔ19 mutant did replicate, but this was delayed until 50 days post-infection, only common prior to which pUΔ19 culture supernatants were consistently RT negative. The appearance of this phenotype was reproducible, due to compensatory reversion. Conversely, both the pUΔ14 and pUΔ6 variants, involving deletions in nucleotide regions +443-456 and +457-463, respectively, exhibited only minor delays in viral replication (Fig. 5A).

The elimination of sequences downstream of the splice donor (SD) site also resulted in impaired viral replication in the CEMx174 line. The most significant deficits in replication resulted from deletions that removed the entire sequence from just downstream of the SD to the gag initiation codon, i.e. pDΔ60 (Δ+471-530) and, to a lesser extent, pDΔ51 (Δ+481-531). Smaller deletions spanning this region had relatively little impact on replication kinetics. Of these smaller intermediate deletions, only pDΔ19 (Δ+511-529) resulted in a significant delay in viral replication kinetics (Fig. 5B).

We also evaluated the impact of these deletions on viral replication capacity in PHA-stimulated rhesus PBMCs by monitoring the production of SIV capsid protein (p27) in culture supernatants. Under these conditions our results (Fig. 5C) show that pUΔ39, pUΔ33 and pUΔ19 were severely replication impaired in primary cells. The pUΔ14 and pUΔ6 mutants were not significantly affected and retained replication kinetics indistinguishable from wild type virus. In rhesus PBMC, impaired replication was most apparent in mutants pDΔ60 and pDΔ51 (Fig. 5D), similar to that observed in the CEMx174 line. Slight delays in viral replication were observed for the smaller deletions within this region, with generally similar data obtained from both primary cells and cell lines.

**Figure 6:**
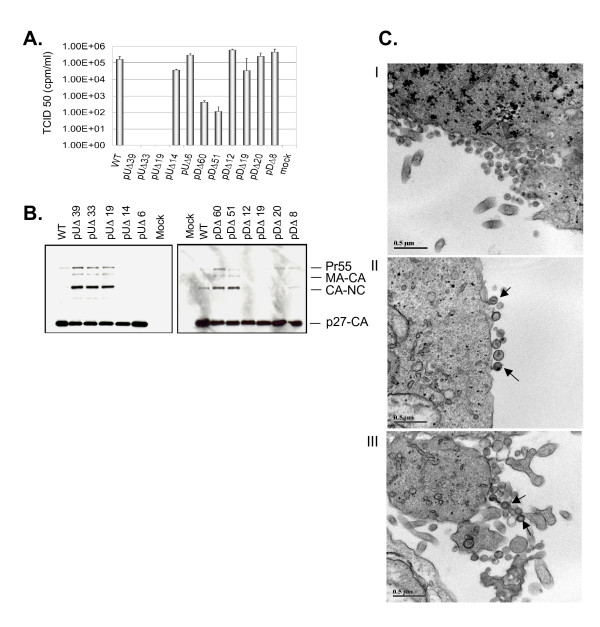
**Analysis of protein expression and viral core ultrastructure of wild type and mutant viral particles**. **A**. Viral replication analysis of mutated viruses TCID_50 _analysis of viral infectivity, scale of ordinate is logarithmic. **B**. Western analysis of wild type and mutant virus particles **C**. TEM of late (fixed 36 hr post-transfection) wild type and mutant particles were assessed and scored from multiple sections. Panel **I**, wild-type virus has typical size and core morphology. Panel **II**, the pUΔ19 mutant shows diminished production of viral particles, with altered core placement and morphology. Panel **III**, shows the pDΔ60 mutant; virus particle release less affected; particle condensation to a mature state is impaired. Bar size is .5 μM.

The infectivity of these mutants was assessed using the endpoint dilution method (TCID_50_) in CEMx174 cells (see Materials and Methods). The results of Fig. 6A show that infectiousness of viral leader mutants was impaired by mutations on both sides of the major SD. Deletions between nucleotide +424 to +462 had the most dramatic effect on viral infectivity, resulting in a >3 log reduction for the mutants pUΔ39, pUΔ33 and pUΔ19 compared with wild-type infectivity. Consistent with the replication experiments described above, the mutant pUΔ14 displayed insignificant reductions in viral infectivity and pUΔ6 was unaffected.

In the region downstream of the SD, (i.e. nt +471 to +530), the mutants' pDΔ60 and pUΔ51 exhibited moderately diminished infectiousness. Again, consistent with that described above, the mutant pDΔ19 displayed a modest but significant reduction in infectivity, while the remaining mutants were not compromised.

To determine the impact of deletion mutagenesis within the SIV leader region on gag expression, particle formation, and viral maturation, COS-7 cells were transfected with either wild type or mutant constructs. Virus containing culture supernatants were first clarified then pelleted by ultracentrifugation through a 20% sucrose cushion.

The Western analysis of upstream leader mutants revealed that total production of mature p27-CA protein was severely diminished in the mutants deleted of leader regions both up and downstream of the SD. This appeared to be the result of a block in the proteolytic processing of Pr^55^Gag protein. This phenotype was prominent in the Western analysis of deletions within SL1 (pUΔ39, pUΔ33 and pUΔ19), and was manifest as an accumulation of the first and second cleavage intermediates (shown in Fig. 6B). Similar results were observed for the pDΔ60 and pUΔ51 mutants, both displaying an accumulation of intermediate processing products.

To further characterize this phenotype, we examined cell-free virus particles by transmission EM (TEM) 36 hrs after the transfection of COS-7 cells with wild type or mutant proviral constructs. Multiple sections of stained cell preparations were scanned by TEM and approximately 100 particles were scored for each virus mutant (see Fig. 5C). Wild-type constructs (Fig. 6C, panel I) resulted in the production of particles of typical morphology and dimension (≈ 100 nm average) and condensed conical cores were observed in >85% of particles with the remainder being of immature morphology.

In contrast, both pUΔ19 and pDΔ60 showed diminished particle production in conjunction with abnormalities in the viral core (Fig. 6C, panel II and III respectively); these were represented as either an immature-like core phenotype or grossly atypical core morphology.

## Discussion

We have demonstrated that RNA elements on both sides of the SD are critical to the viral lifecycle. Not only are these specific elements required for both the efficiency and specificity of vRNA encapsidation, both are important in regard to the dimerization of vRNA. We have shown that these vRNA deficits are linked with perturbations in Pr^55^Gag processing, which ultimately disrupt viral core architecture resulting in diminished viral infectivity and replication.

Studies describing RNA packaging in HIV-1 or HIV-2 have shown that the 5' proximal stem of SL1 interacts directly with NC protein during the encapsidation process [28,37]. However, deletions removing the upstream side of the DIS stem, from both HIV-1 and SIV mutants, resulted only in modest replication deficits compared to the severely impaired replication of mutants harboring nucleotide deletions to the distal side of SL1. In this study, not only did the SL1-pUΔ19 mutant display more than an 84% reduction in packaged vRNA but the comparative decrease in infectivity was greater than 100-fold. Similar observations have been reported regarding deletion of the equivalent region of HIV-1 (Δ+424-442) [28].

Although other groups have reported that the region flanking SL1 (between SL1 and the SD) harbors the critical upstream packaging determinant, our results show that SL1 acts as the primary *cis*-encapsidation determinant. Discrepancy may be due to the specific nucleotide deletions that were generated, or partially reflect the different methods used. However, as mentioned, we used two independent methods to confirm packaging in addition to confirmation by non-denaturing Northern analysis. Importantly, the analysis of viral mutants harboring deletions proximal to SL1 (pUΔ14 and pUΔ6) had no significant effect on viral infectivity or *de novo *replication kinetics in CEMx174 lines or in rhesus PBMC. Yet, the pUΔ14 mutant was found to reduce vRNA packaging by approximately 40% perhaps indicating that the secondary structure of this element may be more critical than the loss of primary sequence in regard to its function. Thus, the primary role of this element may be as a part of an extended DLS structure, rather than solely as a *cis*-packaging element.

In regard to vRNA packaging, the nucleotide region downstream of the SD appears to play a secondary role relative to regions located upstream of the major SD. The most dramatic defects on packaging were observed when the entire terminal 3' leader sequence was deleted (in the mutant pDΔ60 or pDΔ51). Surprisingly, even these large deletions failed to completely abrogate genome packaging in SIV_mac_, contrary to the discrete packaging elements described for simple retroviruses [2]. We used a series of smaller targeted deletions to establish a specific role for each of the stem-loop elements downstream of the SD. We found that packaging specificity could be ascribed in a roughly additive fashion to the smaller deletions disrupting either the SL3 or SL4 element.

Collectively, these data show a multipartite contribution of RNA-leader sequences in SIV vRNA encapsidation, similar to HIV-1. However, the importance of these domains in regard to vRNA packaging appears to be reversed in orientation compared to SL1 and the Ψ core of HIV-1 [2,4]. While RNA domains on both sides of the SD act in concert to selectively incorporate full-length genomic transcripts, we have ascribed the largest single contribution to SL1, whereas both SL3 and SL4 together provide a significant degree of specificity. Accordingly, we have denoted SL1 (Ψ1) as the major determinant and SL3 and SL4 as secondary elements (Ψ2, Fig. 1B). In parallel with HIV-1, it is probable that accessory packaging elements exist elsewhere within the untranslated leader region and possibly within other coding regions as well [2]. For instance, the presence of a functional intron within the SIV/HIV-2 R-U5 region of the UTR, a novel element for lentiviruses, could afford an additional level of regulation of packaging of genomic length RNA [21,38]. The presence of packaging determinants within coding regions has also been established for other retroviruses [2].

In regard to RNA dimerization, our non-denaturing Northern analysis has shown that the leader regions involved in dimerization are immediately proximal to the major splice donor. The results presented here are in agreement with the previous studies of HIV-1 [24,25,39] and HIV-2 [32].

Whether vRNA encapsidation in HIV or SIV preferentially occurs with a single vRNA strand or as a "dimer" composed of two full-length vRNA molecules has not been completely elucidated. In the case of MMLV and MLV, recent work has strongly favored the latter notion, and dimerized RNA has been show to be a necessary step prior to encapsidation [40,41]. In the case of HIV-1, the absolute requirement for dimeric vRNA in mature virions may be dispensable as HIV-1 variants containing only single genomes have been shown to be capable of undergoing replication [6,42]. Other studies using SL1 deficient HIV mutants indicated that the necessity for intravirion dimerization may be target cell specific, or facilitated by functional redundancy elsewhere within the viral genome [26,43].

Disrupted phenotypes exhibiting or laterally displaced cores were observed for several of our SIV mutants (Fig. 6). Several studies have supported the notion that viral RNA plays an important structural role in viral core formation and stability during viral budding and morphogenesis [26,44,45]. We have also demonstrated a relationship between viral core formation and deficits in RNA dimerization and altered Pr^55 ^Gag processing, ultimately impacting replication capacity and viral infectivity.

Similar observations have been reported for HIV-1 where alterations of the "normal" vRNA compliment resulted in morphological anomalies [46,47]. Forced evolutionary studies of SL1 deficient variants of HIV-1 have shown a direct role for the DIS/KLD with multiple protein domains of NC, as well as other regions of the Gag-polyprotein [28]. The compensatory reversion of these HIV-SL1 mutants led to wild-type levels of HIV-1 packaging, but not RNA dimerization, and to modified interaction within several regions of Gag, including the p2 protein [5,8,9].

In sum, the elimination of structures on either side of the SD in SIV yields a replication-impaired phenotype, which is attributable to blocks in RNA encapsidation and dimerization.

## Materials and methods

### Construction of recombinant provirus

We used PCR-based mutagenesis and conventional cloning techniques [48] to generate all deletion mutants; Pfu polymerase was used for all PCR reactions. A full-length infectious clone of SIV, SIV_mac239 _was used as a template to construct all deletion mutants [13]. Briefly, the region between the NarI and BamHI sites in SIV_mac239 _was replaced by recombinant PCR fragments to generate all mutant constructs (Figure 1). For construction of the mutants upstream of the major SD, namely pUΔ39, pUΔ33, and pUΔ19, primer pairs pUΔ39/pSgag, pUΔ33/pSgag1, and pUΔ19/pSgag1 (Table 1) were used to generate deletion fragments that were purified, digested with KpnI/BamHI, and ligated with a corresponding EcoRI/BamHI fragment from the wild-type vector. The resulting fragment was digested with EcoRI and BamHI and inserted into the SIV_mac239 _clone. For the construction of mutants' pUΔ14 through pDΔ8, a first round PCR was performed using consecutive primers paired with the primer SU5. For the mutants downstream (pD) of the major SD i.e. pDΔ60 through pDΔ8 (Table 1), a first-round PCR was performed using consecutive primers paired with the primer SU5. The resultant fragments were used as mega-primers paired with the Sgag1 primer to generate the required deletion fragments that were then used to replace the NarI/BamHI fragment from the wild-type clone. The validity of all constructs was confirmed by sequencing. Nucleotide designations are based on published sequences; the transcription initiation site corresponds to position +1.

**Table 1:** Primers utilized in these experiments

Name	Sequence	Location^a^
pUΔ39	5'-gtcggtacca/caggtaagtgcaacac-3'	+1189-1254
pUΔ33	5'-gtcggtacca/cggttgcaggtaagtgc-3'	+1189-1249
pUΔ19	5'-gtcggtacca/gaggaagaggcctccggttgc-3'	+ 1189-1238
pUΔ14	5'-gcacttacctgcaaccg/tcccgctcctcacgcc-3'	+1216-1254
pUΔ6	5'-gtgttgcacttacctg/gaggcctcttcctctcc-3'	+1203-1249
pDΔ60	5'-ggagtttctcacgcccatct/cttacctgcaaccggag-3'	+1230-1326
pDΔ51	5'-ggagtttctcacgcccatctc/gtgttgcacttacctgc-3'	+1238-1326
pDΔ12	5'-cccttcctggataaaagacagc/gtgttgcacttacctgc-3'	+1238-1288
pDΔ19	5'-ggagtttctcacgcccatctc/cttcctggataaaagacagc-3'	+1267-1326
pDΔ20	5'-ccactctatcttattaccc/tatttcttttttgtgttgcacttacctgcaac-3'	+1229-1305
pDΔ8	5'-cctggataaaagacagctatttctttttt/cttacctgcaaccggagg-3'	+1226-1283
Sgag1	5'-gcaaccccagttggatccatctcctgt-3'	+1348-1322
su5	5'-aagctagtgtgtgttcccatctc-3'	+175-197
Senf	5'-ggcttgagctcactctcttgtgag-3'	+8705-8728
Sg	5'-cttccctgacaagacggag-3'	+567-549
sg1	5'-gaagcatgtagtatgggcag-3'	+627-646
sg2	5'-ggcactaatggagctaagaccg-3'	+746-725
su3	5'-ccggaagggatttattacagtg-3'	-518-497
su3-1	5'-ggctggctatggaaattagtccc-3'	-390-367

^a ^Location of the primer is relative to the transcription initiation site (+1)

### RNA slot-blotting and RT-PCR

For analysis by slot blotting, viral RNA was isolated from purified virus recovered from transient transfection assays, clarified, and subsequently purified through a 20% sucrose cushion. Virus pellets were re-suspended in TE buffer and digested with DnaseI to remove potential plasmid contamination. Sample RNA was purified and then analysed as per Northern analysis described below, using the Scheicher and Schuell Minifold II slot-blotting system. The slot-blot analysis of cellular RNA was also conducted in parallel. Briefly, transfected COS-7 cells were washed twice with cold phosphate-buffered saline and lysed with NP-40 lysis buffer [45]. The cellular RNA within lysates was incubated in the presence of proteinase K (100 μg/ml) for 20 min at 37°C in the presence of 50 U of DNAse I. Phenol: chloroform purification consisted of two extractions in phenol: chloroform: isoamyl alcohol, then chloroform. Viral RNA was then precipitated, washed in 70% ethanol and stored at -80°C until required, at which time samples were resuspended in TE buffer at 4°C. RNA was transferred to Hybond-N nylon membranes by vacuum assisted slot blotting using a 20× concentration of sodium chloride, sodium phosphate-EDTA buffer (SSPE). Membranes were baked for 2 hrs at 80°C. Probes were prepared by digestion of the full-length proviral clone of SIV_mac239_[13], and purification of either the NdeI-BstIIE fragment (from U5 to RT region) to assess total virion RNA content, or by, using a BstIIE double digestion fragment (RT region only) to hybridize with genomic viral RNA. Probes were radioactivity labelled with δ-P^32^-ATP by nick-translation following standard manufacturer's protocols (Roche, Indianapolis, IN, USA) and used in standard hybridization reactions. Relative amounts of products were quantified by molecular imaging (BIO-RAD Imaging). RNA encapsidation was determined on the basis of three different reactions, and calculated with wild type virus levels arbitrarily set at 1.0.

To study packaging of viral genomic RNA, viral RNA was isolated using the QIAamp viral RNA mini kit (QIAGEN) from equivalent amounts of COS-7 cell-derived viral preparations (normalized by SIV p27 antigen). RNA samples were treated with RNase-free DNase I at 37°C for 30 min to eliminate potential plasmid DNA contamination; followed by inactivation by incubation at 75°C for 10 min. The viral RNA samples were quantified using the Titan One Tube RT-PCR system (Boehringer Mannheim, Montreal, Quebec, Canada). The primers sg1 and sg2 were used to amplify a 114-bp fragment representing full-length viral RNA. The primer sg2 was radioactively labeled with δ-P^32^-ATP in order to visualize PCR products. Equivalent RNA samples, based on p27 antigen levels, were used as templates in an 18-cycle RT-PCR. The products were fractionated on 5% polyacrylamide gels and exposed to X-ray film. Relative amounts of products were quantified by molecular imaging (BIO-RAD Imaging). RNA encapsidation was determined on the basis of four different reactions, and calculated with wild type virus levels arbitrarily set at 1.0.

### Northern analysis

Culture fluids from transfected COS-7 cells were collected and clarified using a Beckman GS-6R bench centrifuge at 3,000 rpm for 30 min at 4°C. Viral particles were further purified through a 20% sucrose cushion at 40,000 rpm for 1 hour at 4°C using a SW41 rotor in a Beckman L8-M ultracentrifuge. Viral pellets were first dissolved in Tris-EDTA (TE) buffer, then in lysis buffer containing proteinase K (100 μg/ml) and yeast tRNA (100 μg/ml). Samples were incubated for 20 min at 37°C, in the presence of 50 U of DNAse I, followed by two extractions first in phenol: chloroform: isoamyl alcohol, then chloroform. Viral RNA was then precipitated, washed in 70% ethanol and stored at -80°C until required, at which time samples were resuspended in TE buffer at 4°C. RNA was then analysed by non-denaturing electrophoresis on 0.9% agarose gels in 1× Tris-Borate-EDTA (TBE) running buffer for 4 hrs at 4°C. Products were subsequently denatured in 50 mM NaOH and equilibrated in 200 mM Na-acetate. Following electrophoresis, RNA was transferred to Hybond-N nylon membranes by capillary blotting using a 20× concentration of SSPE buffer. Membranes were baked for 2 hrs at 80°C. Probes were prepared by digestion and purification of the NdeI-BstE III fragment excised from the SIV_mac239 _plasmid. These were recovered by gel purification and labelled with δ-P^32^-ATP by nick-translation following standard protocols (Roche, Indianapolis, IN, USA). The denaturing Northern analysis of cellular RNA was also conducted in parallel. RNA extraction was carried out similar to that described for slot blotting above. The cellular RNA within extracted from lysates was normalized on the basis of p27-Ca antigen present in cellular lysates. Total cellular RNA preparations, i.e. equivalent volumes of RNA, were also run on 1% ethidium bromide (EtBr) stained gels as an internal control for total RNA and 28S and 18S ribosomal RNAs. Probes were prepared as described above Probes were labelled by nick-translation following standard manufacturer's protocols (Roche, Indianapolis, IN, USA) and used in standard hybridization reactions.

### Cell culture and transfection

COS-7 cells were maintained in DMEM medium supplemented with 10% heat-inactivated fetal bovine serum. All media and sera were purchased from GIBCO Inc. (Burlington, Ontario, Canada). CEMx174 cells were maintained in RPMI-1640 medium supplemented with 10% heat-inactivated fetal bovine serum. Monkey PBMCs were isolated from the blood of healthy rhesus macaques (*Macaca Mulatta*), purchased from L.A.B. Pre-Clinical Research International Inc., Montreal, Quebec. All primates were housed in accordance with accredited laboratory care standards. All donor macaques were serologically negative for simian type-D retrovirus (SRV-1), simian T-cell lymphotrophic virus type-1 (STLV-1), and simian foamy virus (SFV-1) at the time of blood collection.

Primate PBMCs were ficoll-purified followed by phytohemagglutinin (PHA) stimulation for 3 days, then maintained in RPMI-1640 medium supplemented with 10% heat-inactivated fetal bovine serum and 20 U/ml IL-2. Molecular constructs were purified using a Maxi Plasmid Kit (QIAGEN Inc. Mississauga, Ontario, Canada). COS-7 cells were transfected using the above constructs with lipofectamine 2000 reagent (GIBCO, Burlington, Ontario, Canada). Virus-containing culture supernatant was harvested at 48 h post-transfection and clarified by centrifugation for 30 min at 4°C at 3,000 rpm in a Beckman GS-6R centrifuge. Viral stocks were passed through a 0.2 μm filter and stored in 0.5 ml or 1 ml aliquots at -80°C. The concentration of p27 antigen in these stocks was quantified using a Coulter SIV core antigen ELISA assay (Immunotech Inc., Westbrook, ME, U.S.A.).

### Virus replication in CEMx174 cells and rhesus macaque PBMCs

To initiate infection, viral stocks were thawed and treated with 100 U of DNase I in the presence of 10 mM MgCl_2 _at 37°C for 0.5 h to eliminate any residual contaminating plasmid DNA prior to inoculation of cells. Infection of CEMx174 cells was performed by incubating 10^6 ^cells with 10 ng of viral p27 antigen equivalent for 2 hrs at 37°C. Infected cells were then washed twice with phosphate-buffered saline and re-suspended in fresh supplemented RPMI-1640 medium. Cells were split at a ratio of 1:3, twice per week. Supernatants were routinely monitored for virus production by reverse transcriptase (RT) assay.

Virus infectivity (TCID_50_) was determined by infection of CEMx174 cells as previously described [49]. Results were calculated by the method of Reed and Muensch.

Virus replication was also assessed in rhesus PBMCs. Briefly, 4 × 10^6 ^activated rhesus macaque PBMCs were infected with SIV stocks containing 10 ng of p27-CA equivalent at 37°C for 2 hours; the cells were then washed extensively to remove any remaining virus. Cells were maintained in 2 ml of culture medium as described above, and fresh stimulated PBMCs were added to the cultures at weekly intervals. Virus production in culture fluids was monitored by both RT assay and SIV p27 antigen capture assay (Coulter Immunotech Inc., Westbrook, ME, U.S.A.).

### Western analysis of viral protein

At 48 hrs post-transfection, virus containing supernatants from COS-7 transfected cells were collected and clarified at 3000 rpm for 30 min. at 4°C in a GS-6R Beckman centrifuge. Virus was further purified by pelleting through a 20% sucrose cushion by ultracentrifugation at 35000 rpm in a Beckman ultracentrifuge for 1 hr at 4°C. Cells were washed 2× in cold PBS and lysed by the addition of buffer containing 1% Nonidet P-40, 50 mM Tris-CL (pH 7.4), 150 mM NaCl, 0.02% sodium azide, and a cocktail of protease inhibitors (Roche, Laval, Quebec, Canada). Virus was normalized on the basis of p27-CA protein present in supernatants or cell lysates. Both pelleted virus and cellular lysates were subject to Western blotting following standard protocols [45] with monoclonal antibodies directed at SIV p27-CA antigens (Fitzgerald industries, MA USA).

### Electron microscopy

Viral ultra-structure for the described mutant viruses was examined by transmission electron microscopy. Briefly, COS-7 cells transfected with wild type or mutant SIV constructs were fixed 48 hours post-tranfection in 2.5% glutaraldehyde/phosphate buffered saline followed by a secondary fixation of lipids in 4% osmium tetroxide. Samples were routinely processed and serially dehydrated, and subsequently embedded in epon under vaccum. Thin-sectioned samples were stained with lead citrate and uranyl acetate and visualized at 80 KeV using a JEOL JEM-2000 FX transmission electron microscope equipped with a Gatan 792 Bioscan wide-angle 1024 × 1024 byte multi-scan CCD camera.
